# Proceedings of Quincy Medical Society

**Published:** 1866-04

**Authors:** Addison Niles

**Affiliations:** Secretary


### PROCEEDINGS OF QUINCY MEDICAL SOCIETY.

The semi-annual meeting of the Quincy Medical Society was held at the office of Dr. C. A. W. ZIMMERMANN, on the 14th day of November, 1865, at 10 o'clock A.M., Dr. ZIMMERMANN being in the chair.

The following members were present:—Drs. C. A. W. Zimmermann, J. H. Reynolds, E. Hoffman, Wm. Zimmermann, and A· Niles.

Dr. A. J. Miller, being present, was proposed as a candidate for membership, and having complied with the conditions prescribed in the constitution and by-laws, was, by a unanimous vote, admitted as a member.

Dr. Reynolds opened the discussion on spotted fever, and proceeded to detail his observations and experience in the disease. He likewise remarked at length upon its history, pathology, diagnosis, and treatment, but as Dr. R. and other parties to this discussion have not furnished us copies of their remarks, as was expected, we are unable to give them in detail.

On motion, the medical gentlemen present were invited to participate in the discussion, which was continued by Dr. Miller, who spoke upon the cause and pathology of the disease, and made some judicious observations upon its treatment. Dr. Wm. Zimmermann likewise remarked briefly upon the subject. The President closed the discussion with some pertinent observations, giving the latest and most approved opinions of German practitioners upon the pathology and diagnosis of spotted fever.

The Society then adjourned, to meet at 1 o'clock P.M.

The Society convened, pursuant to adjournment, at 1 o'clock P.M., and proceeded to elect a delegate to attend the next meeting of the American Medical Association.

On motion, Dr. Wm. Zimmermann was unanimously elected delegate.

Drs. E. Hoffman, J. H. Reynolds, and C. A. W. Zimmermann were unanimously elected delegates to the State Society, and Drs. G. H. Long, J. T. Fugal, and A. J. Miller substitutes.

The Treasurer reported three dollars in his hands; and the Secretary presented a bill of indebtedness of the Society, amounting to six dollars. This sum was raised by contribution and the debt discharged, leaving in the treasury a balance of three dollars.

On motion, a committee was appointed to present a report of the diseases prevailing in Adams County and its vicinity the present year. The President appointed Drs. Niles, Hoffmann, Reynolds, and A. C. Baker.

The President proposed albuminuria and uræmia as the subjects for discussion at the next meeting. On motion, the President was appointed to lead in the discussion.

Dr. Reynolds read a report of a case of suppuration occurring in the prostate gland of an aged patient, terminating in recevery.

The Secretary reported the case of Michael Clark, aged 13 years, a patient of Dr. L. H. Baker, of this city, whom he visited in consultation with Dr. F. K. Bailey and the attending surgeon, Sept. 8th, 1865. The patient was thrown from a horse about 9 o'clock A.M., causing a compound dislocation of the elbow-joint; the articular extremity of the humerus was thrust through the integuments, and likewise through the sleeve of the shirt two inches, effecting an extensive laceration of the soft parts, a complete rupture of the brachial artery, and an injury of the median nerve. The surgeons in attendance, after careful examination and reflection, and at the same time giving due weight to the adverse opinions which had previously been expressed, decided to attempt to save the arm. The patient having been brought into a state of anæsthesia, by ether, Dr. Baker reduced the dislocation at 3 o'clock P.M., six hours after the accident. The brachial artery was ligated, and three sutures introduced; an ordinary roller bandage was applied; the limb placed in a sling, with cold water dressings, and orders to maintain perfect rest and take quarter-grain doses of sulphate of morphia, repeated, if necessary, every three hours during the night.

Twenty-four hours after the accident, Dr. Baker applied a starch bandage, hoping, thereby, to prevent tumefaction. As reaction occurred, with considerable fever, the following mixture was prescribed:—

℞. Magnesiæ Sulphatis,------------------
Morphia Sulphatis,--------------------
Antimonii et Potassæ Tartratis,------āā gr.ij.

Mice et Signa. A tablespoonful to be taken every three hours.

A sero-sanguinolent discharge commenced to flow from the wound on the second day; on the fifth, it assumed a purulent appearance, and became more free.

The tumefaction, and consequent constriction of the joint, caused so much pain that it became necessary to remove the starch bandage on the fifth day after its application. The swelling of the joint was not excessive, and the pain was at no time severe, except when movement was attempted. The great difficulty in the treatment consisted in keeping the joint quiet. Dr. Baker succeeded in maintaining almost perfect rest of the joint by a modification of Dr. Bond's angular splint, made of tin, with brass arms. This apparatus was applied on the 20th, twelve days after the injury. From this time, no pain was caused by dressing the arm, as the elbow was uncovered about two inches above and below the joint. From this time the case progressed favorably. The apparatus was removed on the sixth week, and a slight motion of the arm was perceptible in the direction of flexion, extension, and rotation. The treatment consisted of water dressings the first few days, and, while the constitutional disturbance continued, the saline and antimonial mixture before mentioned, with morphia when necessary to cause sleep.

The constitutional disturbance was not great, the pulse ranging from 92 to 112. The appetite was tolerably good most of the time, and, after the application of the confining apparatus, the patient was able to take daily exercise about the city.

A small collection of pus on the outer aspect of the joint required puncturing, which was done on the 24th of September. On the 11th of November, the ligature of the artery was removed. Since the date of this report, two small pieces of exfoliated bone passed from the orifices about the joint. On the 25th of January, the last orifice closed. The patient can move the arm about two inches in the direction of flexion and extension; the rotary motion is more limited; the ability to move the arm is increasing; and it is probable that its usefulness will, to a great extent, be preserved.

The Secretary likewise reported the following cases, which occurred in his own practice:—

CASE I. Oct. 4th, 1865, at 11 o'clock A.M., was called to visit Mrs. B., aged 30 years, and found her suffering from excruciating pain in the abdomen, causing her to make an outcry which was heard before entering the house. She had, likewise, a rapid and laborious respiration, with no febrile action. At 9 A.M., she commencee using a vaginal injection composed of one tablespoonful of alum dissolved in one pint of water.

The pain began as the fluid first passed from the syringe, and continued without mitigation. Injected, hypodermically, one-third of a grain of morphia dissolved in one-third of a drachm of water; this, in ten minutes, mitigated the pain and diminished the outcry. In one hour another third was injected, which still further relieved the pain. In one hour after, one-fourth of a grain of morphia was administered internally, and directions given to repeat the dose in an hour or two, if necessary. But, notwithstanding these repeated doses, the pain continued twenty-four hours before it subsided. The patient was directed to keep the bed, and give information if the pain returned.

Oct. 8th.—Was called to visit her again, and found her suffering from inflammation of the uterus. The pain returned two days since, but not being so severe as before, she did not think it necessary to inform her medical attendant. The inflammation yielded in a few days to the application of leeches to the uterus, and suitable doses of morphia to allay the pain.

On a subsequent examination, found the cavity of the uterus to measure, by the sound, three and one-half inches, and the os to be quite open. Her health has not been good since the birth of her last child, now some four years old, and she suffers from pain in the back, side, and lower part of the abdomen, and has leucorrhœa. The uterus is in a state of sub-involution, not having returned to its normal condition since her last confinement. The patent condition of the os explains the ready passage of the fluid into the uterus—the nozzle of the syringe probably entered it. Our female syringes would be safer if the nozzle was made larger, with orifices only in the side of it, to guard against such an accident.

Case II. Oct. 29th, 1865, was called to visit Mrs. C., aged 34. She has not menstruated since June 25th, 1865. About six weeks since, she commenced flowing, which has continued to the present time, with the exception of short intervals. The hemorrhage has, at times, been very profuse for a few hours, then diminishing. She had previously consulted two practitioners, the last of whom decided that she was pregnant, and advised her to submit to no further treatment. Anxious on account of her great losses of blood, and debility, she desired further advice. She is quite anæmic, but her appetite for food is good. Four months have elapsed since her menstruation ceased. She has none of the ordinary symptoms of pregnancy, excepting an interruption of the catamenia and an enlargement of the uterus. The mamma is flaccid, and almost in a state of senile atrophy. No change has occurred either in the color of the areola about the nipples, or in the follicles. The fundus of the uterus is felt midway between the pubis and umbilicus. On vaginal examination, the cervix was found retroverted and harder than usual in pregnancy; the os was somewhat open. The uterus was hard, and in attempting to practise ballotement no evidence of a fœtus could be obtained. My impression was, that there was no living fœtus in the uterus, but, as the patient's condition was critical, and a correct diagnosis a matter of great importance, a consultation was decided upon, and Dr. L. H. Baker was called, when an examination by the stethescope and by the speculum was instituted, the results of which served to confirm the opinion previously entertained. It appearing evident that the life of the patient could not be saved without the removal of the cause of the flooding, an elastic catheter was passed through the os, the distance of four and one-half inches. No serous fluid passed, but blood flowed freely through the catheter. A sponge tent was then introduced into the os, for the purpose of dilating it, in order to ascertain the nature of the uterine tumor. The tent passed into the os with some difficulty, which being overcome, it suddenly went out of sight.

October 30th, 2 o'clock P.M.—Patient has had no flooding since the tent was introduced; it has not passed away, and cannot be found in the vagina. The os was then brought to view with the speculum, and a larger tent introduced.

12 o'clock P.M.—Was called up and requested to visit the patient on account of the severe flooding. On examination, both tents were found in the vagina. The first one had probably found its way into the uterus and penetrated the fibrinous cyst it contained, and was at length expelled by exciting uterine contraction. I at once introduced the tampon, to control the flooding.

Oct. 31, 9 o'clock A.M.—The hemorrhage not entirely stopped. Removed the tampon and introduced a new one. The os more dilated. Prescribed a powder of five grains of ergot, to be repeated every two hours.

4 o'clock P.M.—Removed the tampon and found a fibrinous body in the os. The patient had had active pains since taking the powders; these had become so severe that I suspended them, and replaced the tampon.

9 o'clock P.M.—Was sent for again, on account of severe pain. Removed the tampon and placed a new one. Found the tumor descending. Prescribed fifteen drops of tincture of opium in order to give the patient an opportunity to sleep, and directed the ergot to be given again in the morning.

Nov. 1st, 9 o'clock A.M.—The patient rested some last night. The hemorrhage not entirely ceased. Removed the tampon and found a fleshy mass in the vagina, which, by the partial introduction of the hand, was removed. It was pear-shaped, and evidently filled the whole cavity of the uterus. The patient was directed to keep the bed for ten days, and continue the ergot, if necessary, to check the flowing.

This mole was presented to the Society for inspection. It weighs eight troy ounces, and measures four and one-half inches in length, and two and three-fourths of an inch in breadth. The walls of the mole are three-fourths of an inch thick. It has a central cavity containing a fœtus five lines in length and two and one-half in breadth. The umbilical cord is two and one-half lines in length, and is attached to a small body, probably the placenta, which is consolidated with the walls of the cyst, which are, no doubt, an abnormal development of the membranes enclosing the fœtus. The growth of the fœtus must have ceased about the thirtieth day.

Nov. 2d.—The patient has no unusual discharge, and appears quite comfortable. Prescribed one drachm of sulphite of soda, to be taken three times a day, dissolved in water.

Nov. 12th.—No unfavorable symptoms have occurred; the appetite is good, and the strength improving, and no further treatment deemed necessary.

The Secretary, who was appointed essayist at the annual meeting, then occupied one hour in reading a paper on erysipelas. The leading idea maintained and illustrated was, that erysipelas is a blood disease, not necessarily manifesting itself on the skin, but liable to locate internally, and attack simultaneously, or consecutively, various structures of the body.

On motion, the Secretary was directed to prepare for publication an abstract of the proceedings of this meeting for our city papers, and a full report, with all the papers read, for the Chicago Medical Examiner.

On motion, the Society adjourned, to meet in this place on the third Tuesday in May next, at 10 o'clock A.M.

The meeting was believed to be successful in furthering the objects of the Society, the advancement of medical science, and the promotion of harmony and good-will in the profession.

The members, evidently, separated with mutual cordiality and a determination to labor with increased assiduity in the cause of science and humanity.

ADDISON NILES, *Secretary.*

